# Nephroprotective Effects of *Zataria multiflora* Boiss. Hydroalcoholic Extract, Carvacrol, and Thymol on Kidney Toxicity Induced by Cisplatin in Rats

**DOI:** 10.1155/2021/8847212

**Published:** 2021-01-25

**Authors:** Esmaeel Panahi kokhdan, Hossein Sadeghi, Shima Kazemi, Amir Hossein Doustimotlagh

**Affiliations:** ^1^Medicinal Plants Research Center, Yasuj University of Medical Sciences, Yasuj, Iran; ^2^Student Research Committee, Yasuj University of Medical Sciences, Yasuj, Iran; ^3^Department of Clinical Biochemistry, Faculty of Medicine, Yasuj University of Medical Sciences, Yasuj, Iran

## Abstract

**Background:**

Cisplatin (Cis) is an anticancer drug; however, it has dose-dependent renal toxicity. The current study aims to investigate the protective effects of *Zataria multiflora* Boiss. hydroalcoholic extract (Z.M.B), carvacrol, and thymol on cisplatin-induced nephrotoxicity in rats.

**Materials and Methods:**

Forty-two Wistar male rats were randomly allocated into six groups (*n* = 7). Group I received normal saline; group II received Cis (7 mg/kg. ip); group III received the Z.M.B extract only (500 mg/kg/d, po); group IV received Z.M.B extract (500 mg/kg/d, po) + Cis; group V received carvacrol (50 mg/kg/d, po) + Cis; and group VI received thymol (50 mg/kg/d, po) + Cis. The levels of biochemical markers, oxidative stress parameters, and histopathological staining were determined in serum and renal tissues. Also, the chemical compositions (carvacrol and thymol) of the Z.M.B extract were assayed by HPLC analysis.

**Result:**

The results revealed that Z.M.B extract, carvacrol, and thymol markedly decreased the renal index as compared with the Cis-only group. Also, carvacrol and thymol significantly reduced the blood urea nitrogen level as compared with the Cis-only group. Furthermore, Z.M.B extract, carvacrol, and thymol significantly attenuated the Cis-induced increase in malondialdehyde and nitric oxide metabolite. Additionally, histopathological examination showed that Z.M.B extract, carvacrol, and thymol markedly ameliorated Cis-induced renal tubular necrosis.

**Conclusion:**

The results showed renoprotective effects of Z.M.B extract, carvacrol, and thymol in Cis-induced nephrotoxicity in rats. Therefore, Z.M.B extract can be considered as a potential candidate for the protection of nephrotoxicity induced by Cis.

## 1. Introduction

Cisplatin (Cis) as the short form of cis-diaminedichloroplatinum is one of the major effective drugs used in chemotherapy for a variety of cancers including testicular, ovarian, and cervical cancers [1]. However, nephropathy is a serious complication of this drug, which makes it necessary to reduce the dose of this drug. A number of cases of acute renal failure in hospitalized patients are due to the unavoidable prescription of Cis [2], and despite the hydration therapy as a way to reduce renal toxicity, about one-third of patients who received Cis would experience irreversible kidney injuries [3]. The free Cis in the blood is easily filtered by glomeruli and enters the tubular cell through a transfer process. The main route for the transfer of Cis is active in renal transfer cells; although some amount of Cis also enters through the process of simple release, the kidney collects more Cis than any other organ and is the main route for its excretion [4]. Pathological changes in Cis-induced renal toxicity occur in proximal tubules because they accumulate the highest amount of Cis [5]. The intracellular effects of Cis include mitochondrial damage, cessation of the cell cycle, disruption of cellular transport systems, as well as induction of apoptosis or necrosis [6]. Cisplatin produces free radicals and activates the cascades of cell death signaling; then, it disrupts the antioxidant defense system [7]. Our previous study showed that antioxidant compounds such as *Stachys pilifera* Benth. extract had a protective role against cisplatin-induced renal toxicity [8].

Medicinal plants or their active ingredients are widely used in the treatment of diseases. Several types of medicinal plants have been reported to reduce the toxicity of Cis [9]. *Zataria multiflora* Boiss. (Z.M.B) is a well-known medicinal plant of the Lamiaceae family. In traditional medicine, this plant is used as an antispasmodic and antitussive drug, as well as it is used in the treatment of lung infection and influenza. Carvacrol and thymol are among the important compounds in Z.M.B that are also are responsible for 70% of Z.M.B essential oil. The presence of these compounds in Z.M.B accounts for its high antioxidant properties [10, 11]. In addition, Z.M.B has shown significant protective and inhibitory properties against tissue damage caused by cellular oxidants [12]. Therefore, the current study was designed to investigate the effects of Z.M.B extract and its two main ingredients, carvacrol and thymol, on Cis-induced toxicity in rats.

## 2. Materials and Methods

### 2.1. Plant Materials

*Zataria multiflora* Boiss was collected in the spring of 2019 from Dena Mountain in Yasuj, Iran. The plant was approved by a botanist (herbarium number; Hyu-855-37687). Their aerial parts were dried in the shade away from direct sunlight. The leaves were separated from the stem and were ground and powdered.

### 2.2. Extract Preparation Method

The extraction was performed with maceration technique in which 200 g of plant leaf powder was soaked in 1 L of 75% ethanol at room temperature for 48 hours. This practice was repeated three times. The extract was concentrated in vacuum and dried at 40°C in an incubator. The dried extract was stored at −20°C [13].

### 2.3. Animals

Forty-two rats with a weight range of 180 to 220 g were provided from the Pasteur Institute of Iran and were kept in 12 hours of light/12 hours of darkness and a temperature of 23 ± 2, and they were fed for a week. The study was approved by the Ethics Committee of the Yasuj University of Medical Sciences (Code: IR.YUMS.REC.1398.083). The animals were monitored according to the Principles of Laboratory Animal Care.

### 2.4. Experimental Design

Cisplatin was obtained from a Chinese pharmaceutical company named MYLAN (1 mg/mL). Carvacrol and thymol were purchased from Sigma Chemical Co. (St Louis, MO, USA) and Merck (Germany), respectively. Cisplatin was administered intraperitoneally at 7 mg/kg body weight on the first day [14]. The hydroalcoholic extract of Z.M.B was dissolved in sterile distilled water. Adult male rats were randomly assigned to six groups (*N* = 7) as follows:

  Group I (normal): recipient of normal saline orally for 7 consecutive days
  Group II (Cis): recipient of normal saline for 7 consecutive days after administration of Cis (7 mg/kg)
  Group III (500 mg/kg extract): recipient of only Z.M.B extract (500 mg/kg) orally for 7 consecutive days
  Group IV (500 mg/kg extract + Cis): recipient of Z.M.B extracts (500 mg/kg) orally for 7 days after administration of Cis (7 mg/kg)
   Group V (carvacrol 50 mg/kg): recipient of carvacrol 500 mg/kg orally for 7 days after administration of Cis (7 mg/kg)
   Group VI (thymol 50 mg/kg): recipient of thymol 50 mg/kg orally 7 days after administration of Cis (7 mg/kg)

On the eighth day of the study, rats were anesthetized with ether and blood was collected from their hearts to measure biochemical markers in the serum. Then, both kidneys were removed from the body and were washed with normal saline. One kidney was placed in 10% formalin for histopathological evaluation, and the other was homogenized in PBS (10 mmol/L, pH 7.4). Kidney homogenate was stored in a refrigerator at −20°C to determine the levels of nitrite oxide (NO) metabolite, malondialdehyde (MDA), the ferric reducing antioxidant power (FRAP), and total thiol (tSH). Changes in their weights were measured on the first and eighth day of the experiment.

### 2.5. Measurements of Biochemical Tests

The concentrations of blood urea nitrogen (BUN) and creatinine (Cr) were determined using commercial kits (Pars Azmoon, Iran).

### 2.6. Measurement of MDA

Malondialdehyde was determined according to the reaction with thiobarbituric acid (TBA) [15]. 250 *μ*L of tissue homogenate or serum was added in 1000 *μ*L of reagent (15% w/v trichloroacetic acid, 0.25 N HCl, and 0.375% w/v TBA), and the optical density was assayed at 535 nm.

### 2.7. Measurement of NO Metabolite

NO metabolite levels were determined in serum and kidney homogenate using the Griess reagent [8]. Sodium nitrite was used as a standard.

### 2.8. Measurement of Total Antioxidant Capacity Using the FRAP Method

This method was based on the ability of tissue homogenate in regeneration of ferric ion (Fe^3+^) to ferrous ion (Fe^2+^) in the presence of tripyridyl-s-triazine (TPTZ). The result was a blue complex TPTZ-Fe^2+^ with the maximum absorption of 593 nm. FeSO_4_ 7H_2_O (0–1000 *µ*mol/L) was used as the standard [16].

### 2.9. Measurement of the tSH Level

Total thiol content was determined using a colorimetric method with minor modifications [17]. 25 *µ*L of tissue homogenate was added to 150 *µ*L of Tris-EDTA reagent in a microtube. After that, 790 *µ*L of absolute methanol and 10 *µ*L of 5,5-dithio-bis-(2-nitrobenzoic acid) were added. The tube was kept at 25°C for 15 minutes, and the absorption was measured at 412 nm.

### 2.10. Histopathological Examinations

After removing the kidneys, the left kidney of each rat was cut in half. Then, the two halves were retained in the 10% formalin solution for a few days. After processing, the tissues were placed in the paraffin and cut into 3 to 4 micrometer slices. The incisions were placed on glass slides and stained with hematoxylin and eosin (H&E) staining.

### 2.11. Chromatographic Conditions

High-performance liquid chromatography (HPLC) was performed on KNAUER liquid chromatography (Berlin, Zehlendorf, Germany) equipped with a micro vacuum degasser, a quaternary pump, a UV-VIS 2550 detector (was set at 220 nm for thymol and carvacrol) and a Zorbax SB-C_18_ column (250 mm × 3.9 mm id, 5 micrometer particle size), and an injection volume of 20 *μ*L. The mobile phase contained acetonitrile-water mixture (45:55 V/V%) with a flow rate of 1.1 ml·min^−1^.

### 2.12. Statistical Analysis

Data were evaluated using the one-way ANOVA test. Tukey's multiple comparison was used to express significant statistical significance. The data were presented as mean ± SEM. *P* ≤ 0.05 was considered statistically significant in all experiments.

## 3. Results

### 3.1. Body Weight Changes

In terms of body weight, the Cis group lost some weight in comparison with the control group (Table 1). However, Z.M.B and carvacrol to some extent reversed the weight loss as compared with Cis-only group.

### 3.2. Effects of Cisplatin on Biochemical Tests and Renal Index

Creatinine, BUN, and renal index in the Cis group indicated a marked increase as compared with the control group (*P* ≤ 0.05). The administration of hydroalcoholic extract of Z.M.B extract, carvacrol, and thymol significantly decreased the renal index in comparison with the Cis group (*P* ≤ 0.05). Treatment with carvacrol and thymol markedly decreased the BUN level as compared with the Cis group (*P* ≤ 0.05). However, the administration of hydroalcoholic extract of Z.M.B had no effect on BUN and Cr in comparison with the Cis group (Table 2).

### 3.3. Assessment of Renal Oxidative Stress Parameters

As indicated in Table 3, serum contents of MDA, FRAP, and NO metabolite markedly augmented in the Cis group in comparison with the control group (*P* ≤ 0.05), while treatment with hydroalcoholic extract of Z.M.B, carvacrol, and thymol significantly reduced serum levels of NO metabolite and MDA as compared with the Cis group. However, the FRAP content did not significantly change in the treated groups in comparison with the Cis group (Table 3).

The present study showed that Cis increased oxidative stress parameters such as NO metabolite and MDA, while it reduced the tSH level in renal tissue as compared with the control group (Table 4). The administration of Z.M.B extract, carvacrol, and thymol significantly reduced the NO metabolite level in comparison with the Cis group; however, only carvacrol and thymol significantly reduced the MDA level as compared with the Cis group. The tSH content in the treated groups did not change significantly compared with the Cis group (Table 4).

### 3.4. Histopathological Studies

**Figure 1:**
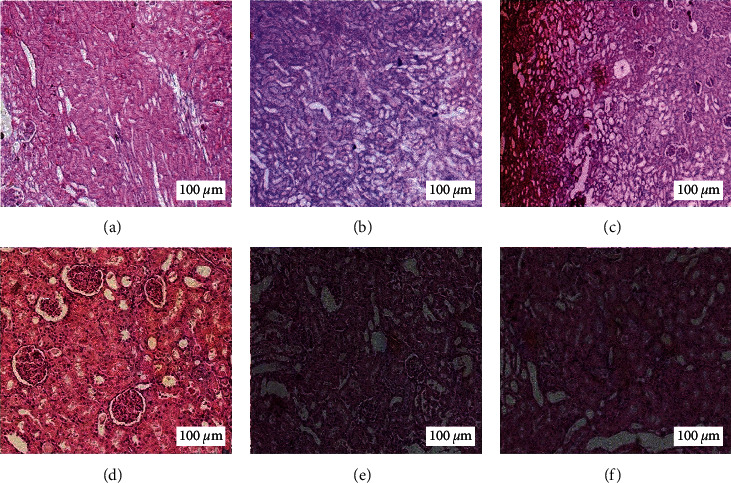
Histological changes in the renal tissue: (a) control, (b) cisplatin (Cis), (c) *Zataria multiflora* Boiss. extract (Z.M.B) only, (d) Z.M.B + Cis, (e) carvacrol + Cis, and (f) thymol + Cis (hematoxylin and eosin, ×100).

According to Figure 1, histological results in the control group showed that the appearance of the kidneys, glomerulus, and urinary tubes were normal. In the Cis group, the size of the kidneys was larger, and the proximal, distal, and urethral tubules were dilated, indicating the destruction of glomeruli, necrosis of the urinary tract, and the infiltration of inflammatory cells. In the group that received both Z.M.B extract and Cis, the destruction of glomeruli decreased to some extent, the bleeding points were decreased, and the size of the kidneys was smaller than only the Cis group, but edema was observed. In the carvacrol and thymol groups, the damages caused by Cis were largely healed and were similar to those of the control group.

### 3.5. Chromatographic Analysis

**Figure 2:**
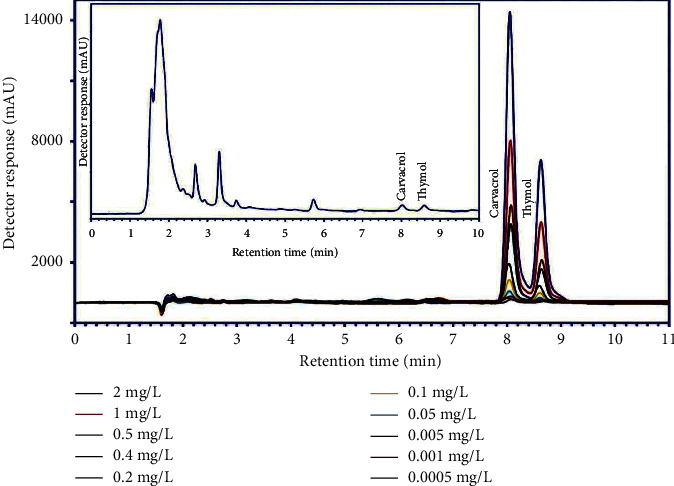
Chromatogram standard samples of thymol and carvacrol in distilled water at different concentrations and the hydroalcoholic extract of *Zataria multiflora* Boiss (inset in the figure).

The concentration of thymol and carvacrol compounds in Z.M.B extract was determined using the standard method. The calibration curve (Figure 2) was plotted according to the concentration (0.0005 to 2 mg·L^−1^), and the unknown concentration of each analyte was calculated. Based on the chromatographic data and standard increase method, the amount of thymol and carvacrol in Z.M.B extract were 101.69 and 81.70 mg·g^−1^, respectively (inset of Figure 2).

## 4. Discussion

The result of the present study revealed that the hydroalcoholic extract of Z.M.B and its two main ingredients, thymol and carvacrol, reduced the renal toxicity induced by Cis in rats. The main limiting factor for the clinical administration of Cis is its renal toxicity. Despite the constant control of serum concentrations during the administration of Cis, dose-dependent nephrotoxicity is still observed in patients [18, 19].

Cisplatin-induced nephrotoxicity appears mainly for at least 3 days after the beginning of treatment, and its specific effects comprise a decrease in the glomerular filtration rate, an increase in serum creatinine and BUN levels, and a decrease in the ability of the kidneys for urine concentration. In addition to all these, proteinuria and electrolyte imbalance also occur [20–24]. Currently, much research has been done to understand the possible protective mechanisms that work against Cis toxicity and attempts have been made to clarify how it works [25].

Reactive oxygen species (ROSs) play a vital role in causing acute renal failure due to the administration of Cis [26]. It is not surprising; therefore, many antioxidants, such as vitamins C and E, can reduce Cis-induced nephrotoxicity [27]. In addition, N-acetyl cysteine has been shown to reduce renal toxicity of Cis in laboratory animals and humans [28].

Medicinal plants show favorable effects against different nephrotoxic agents. Z.M.B extract contains known antioxidants such as carvacrol, thymol, and menthol. It seems that the antioxidants in Z.M.B (carvacrol and thymol) have been able to reduce the relative impairment of kidney function by reducing the amounts of ROS. Our research has shown that rats' weights reduced in the Cis group in comparison with the control group. However, the hydroalcoholic extract of Z.M.B, and carvacrol to some extent, reversed the weight loss caused by Cis.

Our findings confirmed the administration of cisplatin (7 mg/kg body weight) leads to acute renal failure by increasing kidney function tests and histological findings.

The nephroprotective effect of Z.M.B may be related to its inhibitory activity on the secretion or function of inflammatory cytokines and pro-oxidants, which are involved in the damage of glomerular filtration rate [29]. Ibrahim et al. showed that a covalent bond is formed between platinum and some DNA bases, which can lead to cytotoxicity [30].

The present work indicated that the injection of Cis markedly increased the levels of MDA and NO metabolite in renal tissue and serum as compared with the control group. Several investigations have reported that Cis caused a significant elevation in the kidney MDA and NO metabolite concentration [31, 32]. Indeed, the increase of MDA and NO metabolite in the Cis group was lowered by Z.M.B, carvacrol, and thymol. Therefore, the beneficial action of Z.M.B, carvacrol, and thymol on MDA and NO metabolite contents might be due to its antioxidant effects.

HPLC analysis indicated that Z.M.B extract contains phenolic compounds such as thymol and carvacrol. It seems that these phenolic compounds protect tissue against oxidative stress by scavenging free radicals, reducing NO metabolite, and inhibiting lipid peroxidation.

In the current study, tSH groups were reduced following Cis administration. Z.M.B- and thymol-treated rats showed higher tSH levels rather than Cis-only group, demonstrating that Z.M.B and thymol insignificantly accelerated the replenishing of the total thiol pool. The effect of Z.M.B and thymol on total thiol contents may be attributed to its direct antioxidant activity [33].

## 5. Conclusion

The present study supports the role of oxidative stress in the pathophysiology of Cis-induced nephrotoxicity. Hydroalcoholic extract of Z.M.B and its two main ingredients (carvacrol and thymol) were able to improve oxidative stress and kidney damage caused by Cis. However, more studies are required to clarify mechanisms involved in Z.M.B action on Cis-induced nephrotoxicity.

## Figures and Tables

**Table 1:** Effects of Z.M.B on body weight in rats before and after treating with cisplatin (in grams).

Treatment group	Before treatment (*W*_0_)	After treatment (*W*_1_)	% change (*W*_1_ − *W*_0_/*W*_0_)
Control	221.00 ± 23.18	250.83 ± 22.00	13.50
Cis	186.67 ± 18.78	197.50 ± 1.12	5.80
Z.M.B only	190.00 ± 12.91	218.51 ± 5.54	15.00
Z.M.B + Cis	198.00 ± 13.01	216.33 ± 13.21	9.26
Carvacrol + Cis	165.00 ± 6.06	193.83 ± 9.41	17.47
Thymol + Cis	223.33 ± 23.23	239.50 ± 14.59	7.24

Results are presented as mean ± SEM. Cis: cisplatin; Z.M.B: hydroalcoholic extract of *Zataria multiflora* Boiss.

**Table 2:** Effects of Z.M.B hydroalcoholic extract, carvacrol, and thymol on kidney markers in Cis-induced toxicity in rats.

Treatment group	BUN (mg/dL)	Creatinine (mg/dL)	Body weight/kidney weight index
Control	17.40 ± 0.78	0.48 ± 0.02	0.74 ± 0.05
Cis	29.32 ± 2.76^*∗∗∗*^	0.61 ± 0.03^*∗*^	1.03 ± 0.03^*∗∗∗*^
Z.M.B only	18.68 ± 1.34	0.49 ± 0.02	0.73 ± 0.04
Z.M.B + Cis	22.52 ± 2.26	0.53 ± 0.04	0.84 ± 0.03^#^
Carvacrol + Cis	20.95 ± 1.89^#^	0.5 ± 0.02	0.89 ± 0.04^#^
Thymol + Cis	20.88 ± 0.95^#^	0.57 ± 0.03	0.80 ± 0.03^##^

Values are presented as mean ± SEM. Statistically significant compared with the control group, ^*∗*^*P* ≤ 0.05 and ^*∗∗∗*^*P* ≤ 0.001; statistically significant compared with the Cis group, ^#^*P* ≤ 0.05 and ^##^*P* ≤ 0.01. Cis: cisplatin; Z.M.B: hydroalcoholic extract of *Zataria multiflora* Boiss.

**Table 3:** Effects of Z.M.B hydroalcoholic extract, carvacrol, and thymol on the serum oxidative stress markers in Cis-induced toxicity in rats.

Treatment group	MDA (nmol/L)	NO (*µ*mol/L)	FRAP (*µ*mol/L)
Control	487.17 ± 12.21	0.50 ± 0.02	305.10 ± 34.07
Cis	612.17 ± 16.11^*∗*^	1.20 ± 0.12^*∗∗∗*^	672.43 ± 74.73^*∗*^
Z.M.B only	554.82 ± 14.91	0.50 ± 0.04	361.87 ± 47.77
Z.M.B + Cis	443.38 ± 52.17^##^	0.59 ± 0.09^##^	492.75 ± 72.29
Carvacrol + Cis	419.87 ± 29.92^###^	0.48 ± 0.05^###^	566.68 ± 143.76
Thymol + Cis	449.59 ± 11.43^#^	0.57 ± 0.04^##^	610.35 ± 62.38

Values are presented as mean ± SEM. Statistically significant compared with the control group, ^*∗*^*P* ≤ 0.05 and ^*∗∗∗*^*P* ≤ 0.001; statistically significant compared with the Cis group, ^#^*P* ≤ 0.05, ^##^*P* ≤ 0.01, and ^###^*P* ≤ 0.001. Cis: cisplatin; Z.M.B: hydroalcoholic extract of *Zataria multiflora* Boiss.

**Table 4:** Effects of Z.M.B hydroalcoholic extract, carvacrol, and thymol on the kidney tissue oxidative stress markers in Cis-induced toxicity in rats.

Treatment group	MDA (nmol/g tissue)	NO (*µ*mol/g tissue)	tSH (*µ*mol/g tissue)
Control	71.64 ± 0.91	0.35 ± 0.06	17.30 ± 1.68
Cis	84 ± 1.33^*∗∗*^	0.64 ± 0.02^*∗∗∗*^	10.03 ± 0.24^*∗∗∗*^
Z.M.B only	77.88 ± 3.20	0.31 ± 0.04	13.36 ± 0.95
Z.M.B + Cis	76.28 ± 1.98	0.34 ± 0.03^###^	11.28 ± 0.62
Carvacrol + Cis	72.97 ± 0.94^#^	0.39 ± 0.03^##^	9.85 ± 0.97
Thymol + Cis	72.97 ± 2.81^#^	0.37 ± 0.03^###^	12.20 ± 1.53

Values are presented as mean ± SEM. Statistically significant compared with the control group, ^*∗*^*P* ≤ 0.05 and ^*∗∗∗*^*P* ≤ 0.001; statistically significant compared with the Cis group, ^#^*P* ≤ 0.05, ^##^*P* ≤ 0.01, and ^###^*P* ≤ 0.001. Cis: cisplatin; Z.M.B: hydroalcoholic extract of *Zataria multiflora* Boiss.

## Data Availability

The data supporting the findings of this study are available within the article.
